# Shifting Balancing Selection Underlies an Inversion Cline in Eurasian Blackcap

**DOI:** 10.1002/ece3.73749

**Published:** 2026-06-16

**Authors:** Jun Ishigohoka, Miriam Liedvogel

**Affiliations:** ^1^ MPRG Behavioural Genomics, Max Planck Institute for Evolutionary Biology Plön Germany; ^2^ Friedrich Miescher Laboratory of the Max Planck Society Tübingen Germany; ^3^ Institute of Avian Research Wilhelmshaven Germany; ^4^ Department of Biology and Environmental Sciences Carl von Ossietzky Universität Oldenburg Oldenburg Germany

## Abstract

Divergent selection on chromosomal inversions associated with locally adaptive traits can generate inversion clines. Typical clines span a narrow width of the species' geographic range, with each arrangement maintained at high frequency on its respective side. While balancing selection also maintains inversion polymorphisms, it is not known whether spatial heterogeneity in its parameters, or shifting balancing selection, can generate inversion clines. Here, we study a cline of an 8 Mb‐long inversion inv_12_3 in a widespread seasonal migratory songbird, the Eurasian blackcaps ( *Sylvia atricapilla* ). Unlike typical inversion clines, this gradient spans the entire species range with one arrangement being the minor allele in all populations, and thus cannot be explained by divergent selection. More specifically, the inv_12_3 frequency among continental populations is decreased in populations with shorter migratory distance, with residents being the lowest; and the frequency is even lower in six island populations, which are all resident. Using demography inference, population genomic simulation under the blackcap demography, and approximate Bayesian computation, we identify the type of balancing selection and estimate its spatially varying parameters. We show that inv_12_3 is under negative frequency‐dependent selection (NFDS) with a lower optimal frequency on islands. Corroborating with biogeographic history, our results suggest that inv_12_3 was maintained at an optimal frequency in a glacial refugium, then populations diverged through post‐glacial expansion, during which the selection may have changed upon island colonisation. To our knowledge, this is the first case of NFDS suggested to underlie inversion clines, highlighting the underappreciated role of shifting balancing selection.

## Graphical abstract

We investigated what factor contributes to a cline of a chromosomal inversion of the Eurasian blackcap, which differs from typical inversion clines maintained by divergent selection. Using a simulation‐based machine learning approach, we found that the inversion is under negative frequency‐dependent selection, and the optimal frequency is shifted downwads in multiple island populations compared to the ancestral continental population. This result highlights the underappreciated role of shifting balancing selection on inversion clines.

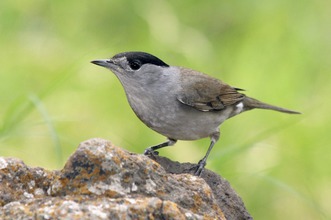

## 1 Introduction

Chromosomal inversions, a structural mutation with a reversed chromosomal segment, can have a major impact on ecological adaptation. Since the pioneering work by Dobzhansky (1947) in *Drosophila melanogaster* , it has been shown that the frequency of inversions in populations of multiple species can be distributed along a geographic axis (Lowry and Willis 2010; Ayala et al. 2013; Kapun et al. 2016; Hager et al. 2022; Nosil et al. 2023). Spatially varying selection due to environmental heterogeneity has been considered to play an important role in forming and maintaining such inversion clines (Kirkpatrick and Barton 2006; Ayala et al. 2013; Kapun et al. 2016; Hager et al. 2022). Inversions often confer large phenotypic effects on multiple traits, and divergent selection on such traits acts as spatially varying selection on the underlying inversions, supporting the critical role of spatially varying selection on inversion clines (Kapun et al. 2016; Todesco et al. 2020; Huang et al. 2020; Hager et al. 2022; Sanchez‐Donoso et al. 2022). In addition to spatially varying selection, which can be regarded as a type of balancing selection in species with geographic subpopulations, many inversions, often without known phenotypic effects, are under some kind of balancing selection and maintained polymorphic over a long period of time (Faria et al. 2019; Berdan et al. 2023). Parameters of balancing selection could also vary across populations in a process called “shifting balancing selection” to form spatial structure of the frequency of inversions (Nosil et al. 2023) or traits (Takahashi et al. 2011).

Despite the evolutionary and ecological importance of selection maintaining polymorphic inversions and its spatial heterogeneity, two questions have been left unanswered in the majority of empirical studies, especially when the phenotypic effect of the inversion is unknown. First, which type of selection maintains the focal inversion polymorphism: (1) spatially varying selection (through divergent selection on traits under genetic control of the inversion); (2) overdominance (heterozygote advantage); or (3) negative frequency‐dependent selection (Faria et al. 2019; Berdan et al. 2023)? Distinguishing these different modes of selection is important for the understanding of the mechanisms underlying the formation and maintenance of spatial structure of the inversion polymorphism. However, such characterization has been limited to species in which phenotypic (Kim et al. 2017; Knief et al. 2017; Mérot et al. 2020; Koch et al. 2021, 2022; Hager et al. 2022; Paris et al. 2025) and fitness effects (Chouteau et al. 2017; Kim et al. 2017; Knief et al. 2017; Mérot et al. 2020; Nosil et al. 2023; Paris et al. 2025) can be experimentally measured. Ecological population genomic studies have identified polymorphic inversions with evidence of long‐term balancing selection in different species, yet the mode of selection maintaining the inversion polymorphism is unknown in most cases because their phenotypic and fitness effect are difficult to measure experimentally in wild species (Harringmeyer and Hoekstra 2022; Stenløkk et al. 2022; Knief et al. 2024; Jamsandekar et al. 2024; Pegan and Winger 2025). Second, what are the parameter values of the selection maintaining the focal inversion and how does it vary between populations? Quantitative measurement and inference of the parameters of spatially varying or balancing selection has been limited both under a controlled condition and in wild populations (Ayala et al. 2013; Hager et al. 2022; Nosil et al. 2023). Furthermore, little is known as to how inversion clines in wild populations are formed as the populations expand or split from their ancestral range to novel environments. One common challenge that makes addressing these questions difficult with population genomics is the confounding by demographic history. Disentangling the effect of selection on inversions from the selectively neutral demography requires joint modelling of selection acting on the inversion and the underlying demography finely tuned to the focal study system (Hager et al. 2022; Nosil et al. 2023).

The Eurasian blackcap ( *Sylvia atricapilla* , “blackcap” hereafter) is one of the most abundant songbird species in Europe (Aymí et al. 2025). Their breeding range spans from Europe to west and central Asia, northern Africa, and Atlantic and Mediterranean islands (Shirihai et al. 2010; Aymí et al. 2025). These widespread populations show geographically structured genetic variation in seasonal migratory behavior (Berthold 1991). Populations breeding at a higher/lower latitude migrate over a longer/shorter distance, with southernmost populations in southern Iberia, northern Africa as well as Macaronesian and Mediterranean island populations being resident (Shirihai et al. 2010; Delmore et al. 2020). Population genomic analyses indicate that these widespread blackcap populations split from an ancestral migratory population tens of thousands of years ago (Delmore et al. 2020; Ishigohoka and Liedvogel 2025). Due to the recent split and large effective population size, blackcap populations show little genome‐wide differentiation (Delmore et al. 2020). Because of their distinct migratory behavior with little genomic differentiation, the blackcap has been an iconic model species for evolutionary genetics of seasonal migration (Liedvogel et al. 2011). Furthermore, their widespread geographic distribution covering a wide variety of ecological conditions including islands makes them a good model species to study genetics of ecological adaptation including the influence of human activity (Pérez‐Rodríguez et al. 2013; Luis Tellería et al. 2013; Andrade et al. 2015; Van Doren et al. 2021).

**FIGURE 1:**
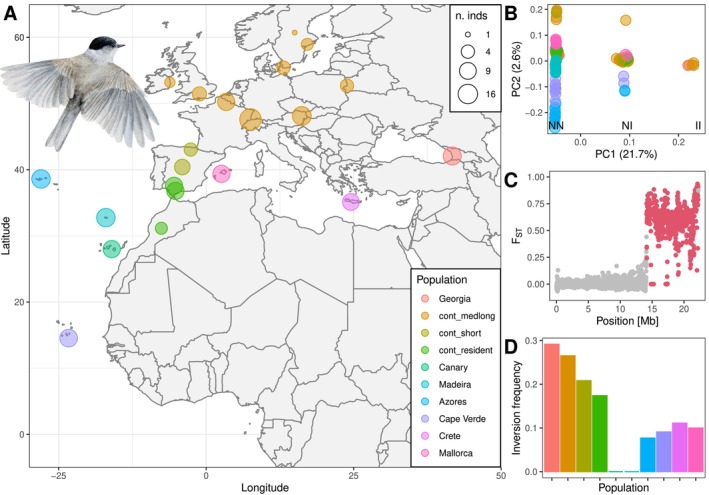
Inversion in blackcaps is under balancing selection. (A) Geographic distribution of blackcap populations included in this study. (B) Local population structure within the 8 Mb‐long inversion of chromosome 12 based on PCA. The three clusters of individuals separated along PC1 correspond to their respective genotype at inv_12_3 (NN: Normal/normal, NI: Normal: Inverted, II: Inverted/inverted). (C) $F_{\mathrm{ST}}$ in 10 kb windows between NN and II along chromosome 12. Red points highlight windows located within inv_12_3. (D) The frequency of the inversion among the blackcap population. Painting by Corinna Langebrake.

We previously identified an 8 Mb‐long inversion polymorphism on the blackcap chromosome 12 (inv_12_3) (Figure 1A; Ishigohoka et al. 2024). The two haplotypes of inv_12_3 are highly diverged compared to the genome‐wide background (Figure 1C), indicating that this inversion polymorphism precedes the population split and has been maintained for a long time (Ishigohoka et al. 2024). Importantly, inv_12_3 has a clinal frequency pattern among populations across their distribution range (Figure 1). Resident populations show consistently lower frequencies of inv_12_3 than migrants, and within residents, the frequency is lower in all island populations than in continental residents. With one arrangement being the minor allele in all populations without known phenotypic effect, the blackcap inv_12_3 cline differs from typical inversion clines with divergent selection that underlies distinct phenotypic variation where one arrangement is nearly fixed in respective side of the cline with one predominant ecotype (Lundberg et al. 2017; Todesco et al. 2020; Huang et al. 2020; Hager et al. 2022; Sanchez‐Donoso et al. 2022). Nevertheless, the long‐term maintenance and the mild yet steady and wide clinal pattern of the blackcap inv_12_3 suggest that it is under some kind of ecologically relevant selection maintaining the polymorphism with spatially varying parameters. In this study, we use population genomic inference and simulation to (1) characterise the mode of selection maintaining the blackcap inv_12_3 and (2) quantify the spatially varying parameters of the selection.

## 2 Materials and Methods

### 2.1 Data and Historical Population Structure

We used phased whole‐genome resequencing (WGS) data of 179 blackcaps available from Ishigohoka et al. (2024). We used Stairway Plot 2 (Liu and Fu 2020) to infer demographic history of each population as a method based on site frequency spectra (SFS) robust to the presence of high‐recombining regions (Ishigohoka and Liedvogel 2025). Specifically, we computed unfolded SFS of chromosomes 1–6 and ran Stairway Plot 2 with default parameters. We also revisited our demography inference with MSMC2 using the low‐recombining half of blackcap genomes in Ishigohoka and Liedvogel (2025), genome‐wide genealogies of blackcaps inferred by Relate using the low‐recombining half of the blackcap genomes from the same study (Ishigohoka and Liedvogel 2025). Both MSMC2 and Relate include cross‐coalescent rate analysis, which can be used to interpret split history of a pair of populations (Speidel et al. 2019; Schiffels and Wang 2020). Of note, for MSMC2, outlier regions based on local population structure (Ishigohoka et al. 2024) including inv_12_3 were removed for the inference (Ishigohoka and Liedvogel 2025). Historical population structure was estimated from genome‐wide genealogies using the RelateCoalescenceRate module of Relate.

### 2.2 Demography Inference With ABC

To select a demography model and estimate demography parameters, we performed approximate Bayesian computation (ABC) with random forest using abcrf (Raynal et al. 2019), and ms (Hudson 2002) as a simulator. We simulated 12 scenarios consisting of the following three factors (1_1_1, 1_1_2, 1_2_1, 1_2_2, 2_1_1, 2_1_2, 2_1_3, 2_1_4, 2_2_1, 2_2_2, 2_2_3, 2_2_4):

i
  1. The population ancestral to the modern Georgian population split earlier than other modern populations.
  2. The population ancestral to the modern Georgian population split with other populations simultaneously.
j
  1. $N_{e}$ of the continental resident population was constant from the ancestral population.
  2. $N_{e}$ of the continental resident population increased.
k
  1. There is no gene flow after population splits.
  2. Gene flow between some pairs of populations is included.
  3. Resident and short distance migrants sequentially split from medium‐long distance migrants without gene flow (only with i=2).
  4. Resident and medium‐long distance migrants sequentially split from short distance migrants (only with i=2).

The demogrpahy parameters were sampled following Table 1. Sampling of parameters and writing of ms commands with sampled parameters for different models were done in custom AWK scripts. In ms simulation, 1000 independent loci with one mutation each were simulated, and the same numbers of individuals as in our empirical data set were sampled from these populations. The ms output was directly piped into an AWK script to compute 75 summary statistics: mean pairwise nucleotide difference π and Watterson's θ for each population, and Weir and Cockerham's estimator of $F_{\mathrm{ST}}$ for all pairs of populations. This simulation was performed 1000,000 times for each of our 12 models. For the empirical data, we randomly sampled 1000 SNPs from autosomes and computed the summary statistics using the same script.

**TABLE 1:** Prior distributions of demography parameters.

Parameter	Explanation	Model	Distribution	Range
$N_{\mathrm{anc}}$	Ancestral population	All	Log uniform	$\left[10^{5},10^{6}\right]$
$N_{\mathrm{geo}}/N_{\mathrm{anc}}$	Relative $N_{e}$ of Georgia	All	Log uniform	$\left[1,100\right]$
$N_{\text{medlong}}/N_{\mathrm{anc}}$	Relative $N_{e}$ of cont_medlong	All	Log uniform	$\left[1,100\right]$
$N_{\text{short}}/N_{\mathrm{anc}}$	Relative $N_{e}$ of cont_short	All	Log uniform	$\left[1,100\right]$
$N_{\mathrm{res}}/N_{\mathrm{anc}}$	Relative $N_{e}$ of cont_res	j=1	1	1
		j=2	Log uniform	$\left[1,100\right]$
$N_{\mathrm{can}}/N_{\mathrm{anc}}$	Relative $N_{e}$ of Canary	All	Log uniform	$\left[0.01,1\right]$
$N_{\mathrm{mad}}/N_{\mathrm{anc}}$	Relative $N_{e}$ of Madeira	All	Log uniform	$\left[0.01,1\right]$
$N_{\mathrm{azo}}/N_{\mathrm{anc}}$	Relative $N_{e}$ of Azores	All	Log uniform	$\left[0.01,1\right]$
$N_{\mathrm{cap}}/N_{\mathrm{anc}}$	Relative $N_{e}$ of Cape Verde	All	Log uniform	$\left[0.01,1\right]$
$N_{\mathrm{cre}}/N_{\mathrm{anc}}$	Relative $N_{e}$ of Crete	All	Log uniform	$\left[0.01,1\right]$
$N_{\mathrm{mal}}/N_{\mathrm{anc}}$	Relative $N_{e}$ of Mallorca	All	Log uniform	$\left[0.01,1\right]$
$T_{\text{split}}$	Simultaneous split time	$k\in \left\{1,2\right\}$	Log uniform	$\left[10^{4},10^{5}\right]$
$T_{\mathrm{geo}}$	Split time of Georgia from ancestral populations	$i=1;k\in \left\{1,2\right\}$	Log uniform	$\left[T_{\text{split}},4\times 10^{5}\right]$
$T_{\mathrm{geo}}$	Split time of Georgia from ancestral populations	$i=1;k\in \left\{3,4\right\}$	Log uniform	$\left[\max\left(T_{\mathrm{pop}}\right),4\times 10^{5}\right]$
$T_{\text{medlong}}$	Split time of cont_medlong from cont_short	i=1;k=4	Log uniform	$\left[10^{4},10^{5}\right]$
$T_{\text{short}}$	Split time of cont_short from cont_medlong	i=1;k=3	Log uniform	$\left[10^{4},10^{5}\right]$
$T_{\mathrm{res}}$	Split time of cont_res from cont_medlong (k=3) or cont_short (k=4)	$i=1;k\in \left\{3,4\right\}$	Log uniform	$\left[10^{4},10^{5}\right]$
$T_{\mathrm{mac}1}$	Split time of ancestral Macaronesian population from cont_medlong (k=3) or cont_short (k=4)	$i=1;k\in \left\{3,4\right\}$	Log uniform	$\left[10^{4},10^{5}\right]$
$T_{\mathrm{mac}2}$	Split time of two subgroups of Macaronesian populations (Can+Mad vs. Azo+Cap)	$i=1;k\in \left\{3,4\right\}$	Log uniform	$\left[10^{4},T_{\mathrm{mac}1}\right]$
$T_{\mathrm{azo},\mathrm{cap}}$	Split time of Azores and Cape Verde	$i=1;k\in \left\{3,4\right\}$	Log uniform	$\left[10^{4},T_{\mathrm{mac}2}\right]$
$T_{\mathrm{can},\mathrm{mad}}$	Split time of Canary and Madeira	$i=1;k\in \left\{3,4\right\}$	Log uniform	$\left[10^{4},T_{\mathrm{mac}2}\right]$
$T_{\mathrm{cre}}$	Split time of Crete from cont_medlong (k=3) or cont_short (k=4)	$i=1;k\in \left\{3,4\right\}$	Log uniform	$\left[10^{4},10^{5}\right]$
$T_{\mathrm{mal}}$	Split time of Mallorca from cont_medlong (k=3) or cont_short (k=4)	$i=1;k\in \left\{3,4\right\}$	Log uniform	$\left[10^{4},10^{5}\right]$
$m_{\mathrm{anc},\mathrm{geo}}$	Symmetrical gene flow between ancestral and Georgia	$i=1;k\in \left\{1,2\right\}$	Log uniform	$\left[10^{-9},10^{-3}\right]$
$m_{\mathrm{geo},\text{medlong}}$	Symmetrical gene flow between Georgia and cont_medlong	$k\in \left\{1,2\right\}$	Log uniform	$\left[10^{-9},10^{-3}\right]$
$m_{\text{medlong},\text{short}}$	Symmetrical gene flow between cont_medlong and cont_short	$k\in \left\{1,2\right\}$	Log uniform	$\left[10^{-9},10^{-3}\right]$
$m_{\text{short},\mathrm{res}}$	Symmetrical gene flow between cont_short and cont_res	$k\in \left\{1,2\right\}$	Log uniform	$\left[10^{-9},10^{-3}\right]$
$m_{\mathrm{res},\mathrm{mac}}$	Symmetrical gene flow between cont_res and all Macaronesian populations	$k\in \left\{1,2\right\}$	Log uniform	$\left[10^{-9},10^{-3}\right]$
$m_{\text{canmad},\text{azocap}}$	Symmetrical gene flow between Can+Mad and Azo+Cap	$k\in \left\{1,2\right\}$	Log uniform	$\left[m_{\mathrm{res}},10^{-3}\right]$
$m_{\mathrm{can},\mathrm{mad}}$	Symmetrical gene flow between Canary and Madeira	$k\in \left\{1,2\right\}$	Log uniform	$\left[m_{\text{canmad},\text{azocap}},10^{-3}\right]$
$m_{\mathrm{azo},\mathrm{cap}}$	Symmetrical gene flow between Azores and Cape Verde	$k\in \left\{1,2\right\}$	Log uniform	$\left[m_{\text{canmad},\text{azocap}},10^{-3}\right]$

To perform model selection, a random forest was constructed using the abcrf function of the abcrf package. Model selection was performed using the predict method of the random forest. Cross validation was performed using the err.abcrf function of the abcrf package. Goodness of fit was visualised using the gfitpca function of the abc package (Csilléry et al. 2012).

To estimate the demography parameters, a regression random forest was constructed using the regAbcrf function of abcrf with a random forest of 1000 trees. Parameters were estimated using the predict method of the regression random forest.

### 2.3 Selection Inference

To select a model of selection on inv_12_3, we performed ABC with random forest using abcrf using SLiM version 4.1 (Haller and Messer 2022) as a simulator. We simulated one neutral model, one model for overdominance, and one model for NFDS. For the overdominance and NFDS models, we included four scenarios. In scenario 1, where all populations have the same parameters, a pair of parameters were sampled (s and h for overdominance and s and $p_{\mathrm{opt}}$ for NFDS). In scenario 2, where parameters differ between migrant and resident populations, two pairs of parameters were sampled and assigned to migrant and resident populations. In scenario 3, where parameters differ between continent and islands, two pairs of parameters were sampled and assigned to continental and island populations. In scenario 4, 10 pairs of parameters were sampled, and they were assigned to different populations. For overdominance, we modelled the genotypic fitness of the three karyotypes as $\left(w_{\mathrm{NN}},w_{\mathrm{NI}},w_{\mathrm{II}}\right)=\left(1,1-\mathrm{hs},1-s\right)$, where s and h are selection coefficient and dominance (Falconer 1989; Walsh and Lynch 2018). For NFDS, we modelled them as $\left(w_{\mathrm{NN}},w_{\mathrm{NI}},w_{\mathrm{II}}\right)=\left(1,1-\mathrm{hs}\left(p-p_{\mathrm{opt}}\right),1-s\left(p-p_{\mathrm{opt}}\right)\right)$, where $p_{\mathrm{opt}}$ is the optimal frequency of the inversion, so that the reduction of fitness is proportional to the frequency deviation from the optimum. For both overdominance and NFDS models, s was sampled from a log uniform distribution between $1\times 10^{-7}$ and $1\times 10^{-1}$. For the overdominance model, h was sampled from a uniform distribution between 0 and −2 (corresponding to the equilibrium inversion frequencies from 0 to 0.4). For the NFDS model, $p_{\mathrm{opt}}$ was sampled from a uniform distribution between 0 and 0.5, while h was held 1/2 for simplicity of the model. Sampling of parameters was done using custom AWK scripts.

We simulated a 1‐bp locus representing inv_12_3 in SLiM version 4.0.1. We scaled the population size and time by a factor of 1000. Assuming that the inversion was at the equilibrium frequency in the ancestral population, we introduced a marker mutation at the equilibrium frequency in the ancestral population (based on sampled h or $p_{\mathrm{opt}}$ for the ancestral population) in the initial generation. For the neutral model, we followed the same procedure as the overdominance model with s=0. This leads to unrealistically high initial frequency for a neutral locus, hence the posterior probability for the neutral model might be overestimated. We implemented a rescaled version of the blackcap population history, and population‐specific fitness effects were implemented within mutationEffect callback based on the genotype of the individual. At the final generation, we sampled the same numbers of individuals as our empirical data set, and recorded the inversion frequency. We ran 1000,000 replicates of simulations per scenario.

We first selected a model (from neutral, overdominance, and NFDS), then selected a scenario (from scenarios 1–4) using the abcrf package. For both steps, we used the abcrf function to construct a random forest. A model and a scenario were selected using the predict method of the random forests. Cross‐validation was performed using the err.abcrf function of the abcrf package. Goodness of fit was visualised using the gfitpca function of the abc package.

To estimate the selection parameters, a regression random forest was constructed using the regAbcrf function of abcrf with a random forest of 1000 trees. Parameters were estimated using the predict method of the regression random forest.

## 3 Results

The study entails two steps. First, we inferred the demographic history of the blackcap populations using whole‐genome SNPs. This inference of neutral demography included identification of a demography model and estimation of demography parameters, which allowed us to simulate selection on inv_12_3 under the inferred blackcap population history in the second step. Second, we characterised selection on inv_12_3 using simulation and observed inversion frequencies. We focused on two possible types of shifting balancing selection: overdominance and negative frequency‐dependent selection (NFDS), both with population‐specific (thus spatially varying) parameters. We excluded the possibility of spatially varying selection across the cline (i.e., selection against and for the inversion in populations connected with gene flow), because the frequency of inv_12_3 stays below 0.5 throughout our sampled populations (Figure 1A,D) which cover most of the species breeding range along the latitudinal axis (Shirihai et al. 2010). We selected among models of neutrality, overdominance, and NFDS, and estimated parameters for the best‐supported model.

### 3.1 Demography Inference Reveal Simultaneous and Independent Split of Populations With Lower Inversion Frequency

**FIGURE 2:**
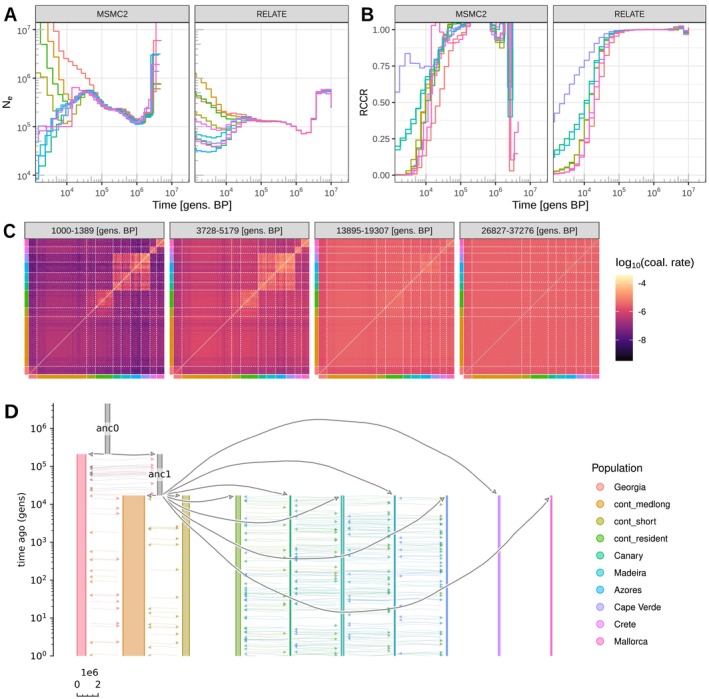
Demography inference. (A) Historical effective population size ($N_{e}$) of blackcap populations inferred with MSMC2 and Relate. (B) Relative cross‐coalescence rate (RCCR) inferred with MSMC2 and Relate for representative pairs of populations. Nine lines show RCCR between Azores and the other nine populations. (C) Historical coalescence rate inferred with Relate. Each 358 × 358 heatmap shows coalescence rates between all 3582=63,903 pairs of haplotypes from 179 individuals at the focal time epoch. The increase in coalescence rate in multiple “clusters” within the heatmap indicates that there were multiple independent isolation events across continental and island resident populations. (D) Demography model inferred through approximate Bayesian computation (ABC) with ABC‐RF. Parts of A and B are reproduced based on dataset from Ishigohoka and Liedvogel (2025).

To characterise demographic history of blackcap populations, we used whole‐genome resequencing data of 179 blackcaps covering their breeding range (Delmore et al. 2020; Ishigohoka et al. 2024). We first used three “model‐free” methods for demography inference: Stairway plot 2 (Liu and Fu 2020), MSMC2 (Schiffels and Durbin 2014; Malaspinas et al. 2016; Schiffels and Wang 2020), and Relate (Speidel et al. 2019). Stairway plot 2 infers effective population size ($N_{e}$) over discretised time for a population based on the site‐frequency spectrum (SFS) and is robust to high recombination rates (Ishigohoka and Liedvogel 2025). MSMC2 approximates the ancestral process along the genome in a structure called ancestral recombination graph (ARG) by sequentially Markovian coalescent (SMC) (McVean and Cardin 2005; Schiffels and Durbin 2014; Malaspinas et al. 2016; Schiffels and Wang 2020). It estimates coalescence rate over discretised time between multiple pairs of sequences from a population or from a pair of populations, which are used to obtain $N_{e}$ for each population and relative cross‐coalescence rate (rCCR) between the population pair over time. Relate estimates the underlying ARG as a series of marginal trees (“genome‐wide genealogies”) based on local distance matrices (Speidel et al. 2019), which are constructed by modelling observed haplotypes and ancestral haplotypes using a hidden Markov model (“chromosome painting”; Li and Stephens 2003). Coalescence rates over discretised time between all pairs of haplotypes are estimated from the inferred genealogies, which can be used to obtain $N_{e}$ and rCCR over time as in MSMC2. MSMC2 and Relate are sensitive to the presence of wide high‐recombining regions in the genome, which is prominent in birds including blackcaps (Ishigohoka and Liedvogel 2025). Stairway plot 2 was run using folded SFS of each blackcap population. MSMC2 was run using a low‐recombining half of the genome of at most four individuals per population. Relate was run using a low‐recombining half of the genome using all individuals. The inferences were qualitatively similar (Figure 2A,B; Figure S1) with consistency in one important aspect of the demography: all resident populations started to split simultaneously.

To further investigate this aspect of population history (independent and simultaneous splits), we used genealogies inferred by Relate to summarise how coalescence rate changed over time (from present to past) for all pairs of haplotypes (Figure 2C). We observed elevated coalescence rates in four distinct clusters of resident individuals: (1) continental resident population; (2) four Macaronesian island populations (Canary Islands, Madeira, Azores, Cape Verde); (3) Mallorca island resident population; and (4) Crete island resident population. These clusters do not merge with each other until the signals disappear (backwards in time). This pattern, in addition to $N_{e}$ and rCCR, indicates that at least four independent split events occurred simultaneously to form both continental and island resident populations. Crucially, all populations in these four groups show reduced frequency of inv_12_3 (Figure 1D). Such consistent shifts in frequency in the same direction are unlikely to be explained purely by neutral drift through demography, indicating that the parameters of balancing selection on inv_12_3 shifted in a consistent manner across all islands and southern end of the continental distribution range (inhabited by continental residents).

We also observed inconsistencies among demography inferences by the three methods in the following three aspects. First, the split of the population ancestral to the current Georgian population was inferred to be older than split events of other modern populations based on Stairway plot 2 (Figure S1) and MSMC2 (Figure 2A,B, left column), whereas Relate inferred that all modern populations including the Georgian population split around the same time (Figure 2A,B, right column). Second, $N_{e}$ of the continental resident population was consistent across the split event according to Stairway plot 2 (Figure S1), whereas MSMC2 and Relate inferred an increase in $N_{e}$ (Figure 2A). Lastly, the estimated values of $N_{e}$ and time differed among these three methods.

To solve these inconsistencies and estimate demographic parameters, we performed approximate Bayesian computation (ABC) using simulations for 12 different models, consisting of combinations of three factors. The first factor was whether or not the split of the ancestral population of the current Georgian population was older than that to other modern populations. The second factor was whether or not the continental resident population increased in $N_{e}$ after the split. The last factor was whether the split of non‐Georgian populations were simultaneous, as well as whether gene flow should be included in the model between some pairs of populations. We included symmetrical gene flow between Macaronesian island populations and between continental populations based on rCCR (Figure 2B) and historical coalescence rate (Figure 2C). By setting parameter ranges covering quantitative differences in $N_{e}$ and time among inferences by Stairway plot 2, MSMC2 and Relate, we covered the space of models and parameters as large as necessary and as small as possible. Each of these 12 models were simulated using ms (Hudson 2002) over 1 million times with randomly sampled parameters. We used abcrf, an implementation of ABC with random forest (Raynal et al. 2019), for model selection. The model_2_1_2 (older Georgian split, constant $N_{e}$ for the continental resident population and non‐zero gene flow) was selected over other models with a posterior probability of 0.621 (Table S1). Parameter inference using abcrf revealed that the ancestral population of the current Georgian population split first ~212,000 generations before present (95% credibility interval (CI) of 41,500–391,000), and other populations split ~17,000 generation before present (95% CI of 10,200–50,600; Table 2).

**TABLE 2:** Parameter estimation of demography model_2_1_2 by ABC‐RF.

Parameter	Expectation	Median	q2.5	q97.5
$N_{\mathrm{anc}}$	$4.08\times 10^{5}$	$4.25\times 10^{5}$	$1.41\times 10^{5}$	$9.50\times 10^{5}$
$N_{\mathrm{geo}}/N_{\mathrm{anc}}$	$2.14\times 10^{0}$	$1.98\times 10^{0}$	$1.03\times 10^{0}$	$6.52\times 10^{0}$
$N_{\text{medlong}}/N_{\mathrm{anc}}$	$5.23\times 10^{0}$	$4.80\times 10^{0}$	$1.17\times 10^{0}$	$3.75\times 10^{1}$
$N_{\text{short}}/N_{\mathrm{anc}}$	$1.36\times 10^{1}$	$1.53\times 10^{1}$	$1.24\times 10^{0}$	$9.18\times 10^{1}$
$N_{\mathrm{can}}/N_{\mathrm{anc}}$	$1.93\times 10^{-1}$	$1.96\times 10^{-1}$	$3.97\times 10^{-2}$	$8.62\times 10^{-1}$
$N_{\mathrm{mad}}/N_{\mathrm{anc}}$	$5.20\times 10^{-1}$	$5.82\times 10^{-1}$	$1.60\times 10^{-1}$	$9.73\times 10^{-1}$
$N_{\mathrm{azo}}/N_{\mathrm{anc}}$	$1.23\times 10^{-1}$	$1.19\times 10^{-1}$	$2.31\times 10^{-2}$	$7.26\times 10^{-1}$
$N_{\mathrm{cap}}/N_{\mathrm{anc}}$	$2.06\times 10^{-1}$	$2.07\times 10^{-1}$	$4.24\times 10^{-2}$	$8.79\times 10^{-1}$
$N_{\mathrm{cre}}/N_{\mathrm{anc}}$	$2.39\times 10^{-1}$	$2.35\times 10^{-1}$	$9.49\times 10^{-2}$	$6.08\times 10^{-1}$
$N_{\mathrm{mal}}/N_{\mathrm{anc}}$	$3.65\times 10^{-1}$	$3.55\times 10^{-1}$	$1.46\times 10^{-1}$	$8.92\times 10^{-1}$
$T_{\mathrm{geo}}$	$1.89\times 10^{5}$	$2.12\times 10^{5}$	$4.15\times 10^{4}$	$3.91\times 10^{5}$
$T_{\text{split}}$	$1.85\times 10^{4}$	$1.70\times 10^{4}$	$1.02\times 10^{4}$	$5.06\times 10^{4}$
$m_{\mathrm{anc},\mathrm{geo}}$	$1.17\times 10^{-7}$	$1.01\times 10^{-7}$	$1.24\times 10^{-9}$	$3.97\times 10^{-5}$
$m_{\mathrm{geo},\text{medlong}}$	$1.82\times 10^{-7}$	$1.98\times 10^{-7}$	$1.27\times 10^{-9}$	$2.74\times 10^{-5}$
$m_{\text{medlong},\text{short}}$	$5.53\times 10^{-7}$	$5.22\times 10^{-7}$	$1.30\times 10^{-9}$	$4.77\times 10^{-4}$
$m_{\text{short},\mathrm{res}}$	$2.05\times 10^{-7}$	$2.22\times 10^{-7}$	$1.28\times 10^{-9}$	$2.80\times 10^{-5}$
$m_{\mathrm{res},\mathrm{mac}}$	$4.49\times 10^{-8}$	$3.38\times 10^{-8}$	$1.16\times 10^{-9}$	$5.06\times 10^{-6}$
$m_{\text{canmad},\text{azocap}}$	$3.25\times 10^{-6}$	$6.23\times 10^{-6}$	$3.63\times 10^{-9}$	$4.39\times 10^{-5}$
$m_{\mathrm{can},\mathrm{mad}}$	$2.98\times 10^{-5}$	$3.51\times 10^{-5}$	$9.70\times 10^{-7}$	$2.09\times 10^{-4}$
$m_{\mathrm{azo},\mathrm{cap}}$	$3.55\times 10^{-5}$	$4.12\times 10^{-5}$	$2.35\times 10^{-6}$	$1.98\times 10^{-4}$

### 3.2 Negative Frequency‐Dependent Selection Shifted in Parallel Across Demographically‐Independent Island Populations

**FIGURE 3:**
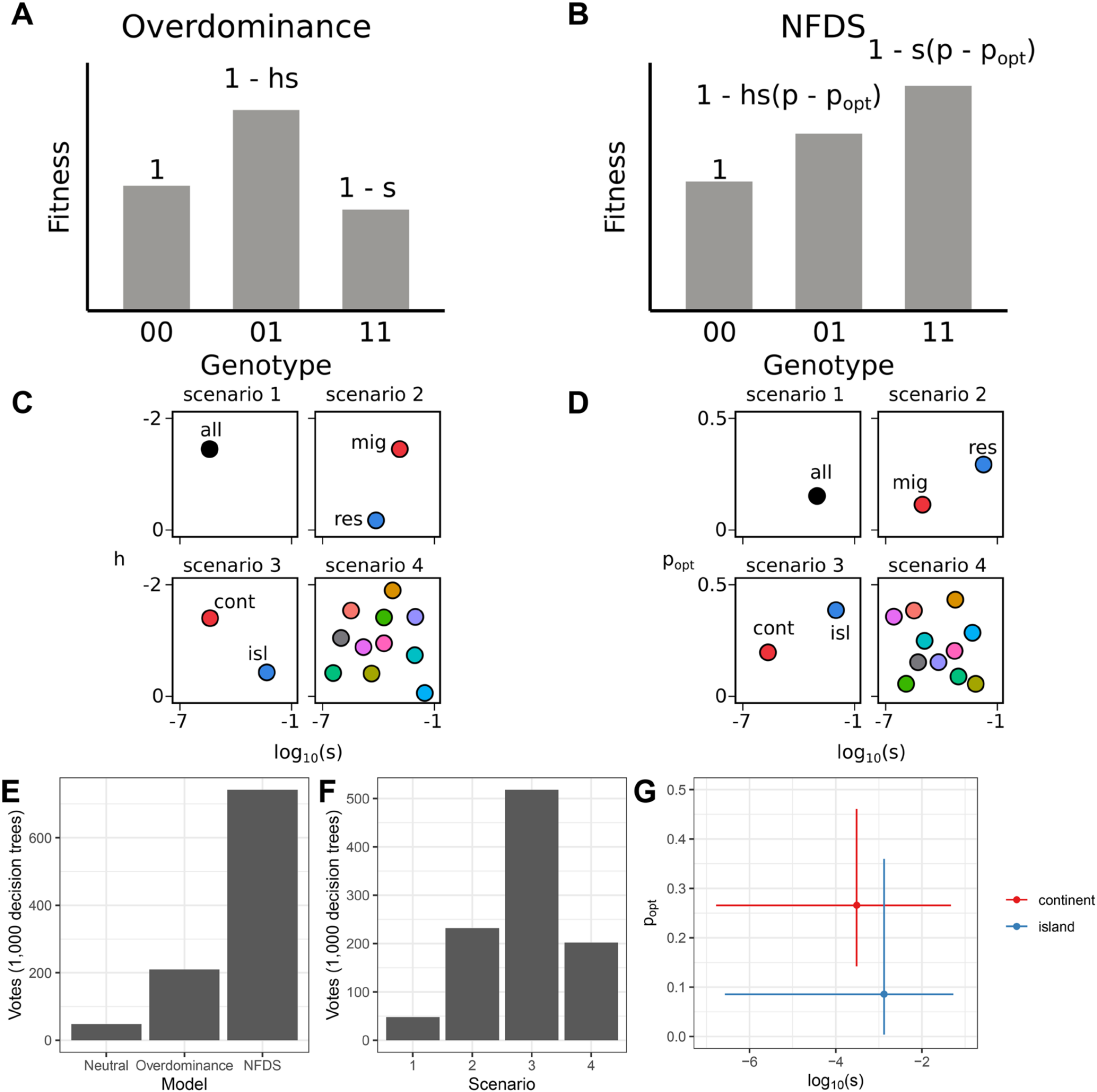
Shifting balancing selection. (A, B) Two candidate models of balancing selection operating on the focal blackcap inversion inv_12_3. In the overdominance (heterozygote advantage) model (A), heterozygotes have higher fitness than homozygotes by the combination of two parameters, dominance (h) and selection (s) coefficients. In the negative frequency‐dependent selection (NFDS) model (B), the fitness of the inversion depends on the difference between the inversion frequency p and the optimal frequency ($p_{\mathrm{opt}}$). For the simplicity of the model, we assumed the effect is additive (i.e., h=1/2) We hypothesise that the lower frequency of the inversion on islands can be explained by different parameters of balancing selection (h and s in overdominance or $p_{\mathrm{opt}}$ and s in NFDS). (C, D) Cartoons showing four scenarios of population difference in overdominance (C) or NFDS (D). In scenario 1, all populations share the same parameter value. In scenario 2, migratory (cont_medlong, cont_short, and ancestral population) and resident populations (cont_resident and all island populations) have different parameter values. In scenario 3, continental and island populations have different parameter values. In scenario 4, all populations have different parameter values. In all scenarios, each parameter was sampled 1000,000 times randomly from a (log) uniform distribution within the range shown in the cartoon. (E) Model selection among neutral, overdominance, and NFDS with ABC‐RF. (F) Selection among four scenarios of NFDS with ABC‐RF. (G) Parameter inference with ABC‐RF. Points show the expectation, and error bars show 95% credibility interval.

We hypothesised that parameters of balancing selection shifted in some populations (island populations or resident populations) by unknown common environmental factors. We tested this hypothesis by addressing two layers of questions. First, we asked what type of balancing selection is acting on inv_12_3 (we term them “models”). Specifically, we asked whether it is overdominance (heterozygote advantage, Figure 3A) or negative frequency‐dependent selection (NFDS, Figure 3B), and we included a neutral scenario as a control. Secondly, we asked in what populations the parameters shifted (Figure 3C,D); we term them “scenarios”. Specifically, we asked whether parameters shifted in resident populations (scenario 2), in island populations (scenario 3), or differently in all populations (scenario 4), in addition to a scenario where parameters do not change (scenario 1).

To select models and scenarios, and to estimate parameters, we simulated selection on a locus for each scenario of each model over one million times in SLiM under the estimated blackcap demography, and performed ABC using abcrf. For each model, we sampled two parameters for simulation: selection coefficient (s) and dominance (h) for model 1 (overdominance); and selection coefficient (s) and optimal inversion frequency ($p_{\mathrm{opt}}$) for model 2 (NFDS). The two layers of questions (models and scenarios) were tested sequentially by two steps of model selection of ABC. For the first layer, the model for NFDS was selected over the other models with a posterior probability of 0.727 (Figure 3E; Table S2). For the second layer, scenario 3 (parameters shifted in island populations) was selected over the other scenarios with a posterior probability of 0.619 (Figure 3F; Table S3). Parameter inference revealed that the optimal frequency $p_{\mathrm{opt}}$ reduced in island populations from 0.266 to 0.086 (Figure 3G; Table S4). This result indicates that the frequency of inv_12_3 reduced in some populations because the optimal frequency of NFDS reduced in island populations.

## 4 Discussion

By modelling demographic history and selection on a polymorphic inversion inv_12_3 of the blackcap, we revealed that (1) negative frequency‐dependent selection (NFDS), instead of overdominance, maintains the polymorphic inversion inv_12_3 in the blackcap, and (2) the optimal frequency of the inversion shifted downwards in island populations compared to the ancestral continental population. As a result of this shift, inv_12_3 is present at consistently lower frequencies in multiple independent island populations compared to continental populations, contributing to the maintenance of the clinal pattern of the inversion frequency over their distribution range. We also showed that most blackcap populations (except for Georgia) split simultaneously at 10,200–50,600 generations before present. This corresponds to 17,300–86,000 year ago, applying a generation time T of 1.70 years based on $T=\alpha +\left(s/\left(1-s\right)\right)$ (α: the age of first breeding, s: adult survival rate (Sæther et al. 2005); for blackcaps α=1 and s=0.441 (Siriwardena et al. 1998)). Placing our demography inference in biogeographic context, the simultaneous split of the populations aligns to the scenario that the ancestral migratory population expanded and diverged through post‐glacial expansion after the last glacial maximum (LGM), in which divergence of migratory phenotypes may have played an important role (Ishigohoka et al. 2026). The exception of the Georgian population having split much earlier than the other populations is also concordant to other animal and plant species which persisted in glacial refugia during the LGM in southern Europe (e.g., Iberia and Balkan) isolated from more eastern refugia, for example, in Caucasus (Taberlet et al. 1998; Hewitt 2000; Neiber and Hausdorf 2015). Although the estimated range of the split time $T_{\text{split}}$ of non‐Georgian populations overlaps not only with the beginning of the Holcene deglaciation (~19,000 years BP) but also with the LGM (20,000–26,000 years BP), an alternative scenario that this split took place during the LGM (e.g., by increased isolation) instead of post‐glacial expansion seems less likely, given the exponential increase in the effective population size of migrants immediately after the split (Figure 2A). Because inv_12_3 was polymorphic in the ancestral population, the expansion after the last glacial retreat may have shifted their inv_12_3 frequency while colonising novel niches. Future investigations of the expansion history of the blackcap populations with spatial population genomics (Osmond and Coop 2024) and ecological niche modelling (Gu et al. 2021) will facilitate further understanding of the ecological and evolutionary drivers forming and maintaining the inv_12_3 cline.

Although the frequency of inv_12_3 shows a continuous gradient across continental populations, we either modelled a discrete shift in the parameters of balancing selection between two groups of populations as a first approximation (scenarios 2 and 3 in Figure 3C,D) or independently sampled the parameters across all populations (scenario 4 in Figure 3C,D). For more fine‐grained modelling of the spatial gradient of the selection parameters, the aforementioned spatial population genomics would be needed, as the geographical and ecological contexts not only vary in space but also have changed over time, which is relevant in the blackcap system.

While the target traits of the NFDS maintaining the inv_12_3 polymorphism are unknown, our results may help narrow down the possibilities. Specifically, good candidates include ecological or social factors that could favour the inversion carrier more when it is rarer (and less so when it is more common), such as frequency‐dependent or disassortative mate preference. For example, female *Drosophila pseudoobscura* has mating preference in a frequency‐dependent manner to males with a rare karyotype (Ehrman 1966), potentially mediated by their ability to differentiate and respond to pheromones of the rare males (Ehrman and Probber 1978). Similarly, in *Heliconius numata*, with a mimicry wing polymorphism controlled by an inversion, females reject males with the same wing morph as themselves (Chouteau et al. 2017). In addition to mate preference, different strategies of defence against predators or parasites could be the target of NFDS (Madsen et al. 2022; Christie and McNickle 2023). Consistently with this possibility, the parasite prevalence in island blackcap populations is lower compared to continental populations (Pérez‐Rodríguez et al. 2013). Interestingly, inv_12_3 contains seven copies of Hydin, which expanded in the songbird lineage (Wirthlin et al. 2014). In mammals, Hydin is expressed in the airway‐lining epithelia (Cindrić et al. 2020), where pathogen and parasites are mechanically removed by mucociliary clearance through coordinated ciliary motility. Because the respiratory ciliary motility depends on Hydin (Lechtreck et al. 2008) and the mechanism of mucociliary clearance is conserved in birds (Maina 2023), the two arrangements of inv_12_3 may affect this anti‐pathogen defence through altered ciliary motility on which NFDS could act differently in island populations. Although the target traits are still elusive, they may well be identified in future studies. For example, one inversion in the zebra finch was found not to be associated with morphological traits or fitness components measured in the initial study (Knief et al. 2016), leaving the target trait of selection unknown. However, later studies identified that this inversion has an overdominance effect on sperm morphology and motility (Knief et al. 2017; Kim et al. 2017) (possibly through difference in spermatogenesis because sperm cells are themselves haploids and therefore cannot be heterozygous). Although quantifying morphological, behavioral, and fitness traits (e.g., parasite prevalence and histological and molecular analysis of respiratory cilia) in wild‐caught animals is not trivial, they will be eventually essential to understand the ultimate cause of the observed cline.

Inversion clines in other systems are often associated with divergent selection on locally adapted traits (e.g., forest and prarie ecotypes in deer mice *Peromyscus maniculatus* (Hager et al. 2022); the “crab” and “wave” ecotypes of marine snails *Littorina saxatilis* (Koch et al. 2021, 2022); adaptation to local host plants in the stick insect *Timema knulli* (Nosil et al. 2023)). In these examples, selection keeps each arrangement (associated with respective ecotype) at high frequency on one side of the narrow cline and low frequency on the other. The blackcap inv_12_3 does not follow this pattern, and we have demonstrated that this is formed by heterogeneity of the selection parameter (optimal frequency) of NFDS acting on the inversion. To our knowledge, this is the first case where NFDS with spatially varying parameter is suggested to generate and maintain an inversion cline (but see Nassar et al. 1973; Kapun et al. 2016). Our finding broadens the theoretical framework of evolutionary mechanisms underlying inversion clines, and raises the question of how common it is that inversion clines are maintained by shifting balancing selection.

## Author Contributions

**Jun Ishigohoka:** conceptualization (equal), data curation (equal), formal analysis (equal), investigation (equal), methodology (equal), visualization (equal), writing – original draft (equal), writing – review and editing (equal). **Miriam Liedvogel:** funding acquisition (equal), project administration (equal), supervision (equal), writing – original draft (equal), writing – review and editing (equal).

## Funding

This work was supported by the Max Planck Society (Max Planck Research Group grant MFFALIMN0001 to ML), and the DFG (German Research Foundation) under Germany's Excellence Strategy—EXC 3051/1 “NaviSense” (project number 533653176 to ML), and as part of project Nav05 within SFB 1372—Magnetoreception and Navigation in Vertebrates (project number 395940726) to M.L.

## Conflicts of Interest

The authors declare no conflicts of interest.

## Supporting information


**Table S1:** Model selection of demography models by ABC‐RF.
**Table S2:** Model selection of balancing selection models by ABC‐RF.
**Table S3:** Model selection of shifting NFDS scenarios by ABC‐RF.
**Table S4:** Parameter estimation of shifting NFDS by ABC‐RF.
**Figure S1:** Demography inference with Stairway Plot 2.

## Data Availability

The scripts used in the study are available at https://github.com/junishigohoka/inv_freq_abc.
