# Supplementary material for: Shifting Balancing Selection Underlies an Inversion Cline in Eurasian Blackcap
Source: Ecol Evol. 2026 Jun 16;16(6):e73749. doi: 10.1002/ece3.73749 (PMC13270080; doi:10.1002/ece3.73749)
Supplement: Supplementary file 1 — Table S1: Model selection of demography models by ABC‐RF. Table S2: Model selection of balancing selection models by ABC‐RF. Table S3: Model selection of shifting NFDS scenarios by ABC‐RF. Table S4: Parameter estimation of shifting NFDS by ABC‐RF. Figure S1: Demography inference with Stairway Plot 2. [file ECE3-16-e73749-s001.pdf]

# Supplementary Information

# Shifting balancing selection on a chromosomal inversion in island populations

Jun Ishigohoka[1,2,*] Miriam Liedvogel[1,3,4,*]

[1]MPRG Behavioural Genomics, Max Planck Institute for Evolutionary Biology, August-Thienemann-Straße 2, 24306 Plön, Germany
[2]Friedrich Miescher Laboratory of the Max Planck Society, Max-Planck-Ring 9, 72076 Tübingen, Germany
[3]Institute of Avian Research, An der Vogelwarte 21, 26386 Wilhelmshaven, Germany
[4]Department of Biology and Environmental Sciences, Carl von Ossietzky Universität Oldenburg, Ammerländer Heerstraße 114-118, 26129 Oldenburg, Germany

[*] Correspondence: Jun Ishigohoka <ishigohoka@evolbio.mpg.de>, Miriam Liedvogel <liedvogel@evolbio.mpg.de>

**Supplementary Table 1:** Model selection of demography models by ABC-RF.

| model | votes |
|---|---|
| 1_1_1 | 0 |
| 1_1_2 | 39 |
| 1_2_1 | 0 |
| 1_2_2 | 15 |
| 2_1_1 | 49 |
| 2_1_2 | 365 |
| 2_1_3 | 136 |
| 2_1_4 | 142 |
| 2_2_1 | 20 |
| 2_2_2 | 168 |
| 2_2_3 | 36 |
| 2_2_4 | 30 |

**Supplementary Table 2:** Model selection of balancing selection models by ABC-RF.

| model | votes |
|---|---|
| neutral | 48 |
| OD | 210 |
| NFDS | 742 |

**Supplementary Table 3:** Model selection of shifting NFDS scenarios by ABC-RF.

| scenario | votes |
|---|---|
| scenario_1 | 48 |
| scenario_2 | 232 |
| scenario_3 | 518 |
| scenario_4 | 202 |

**Supplementary Table 4:** Parameter estimation of shifting NFDS by ABC-RF.

| parameter | expectation | median | q2.5 | q97.5 |
|---|---|---|---|---|
| s_cont | 3.061e-04 | 5.283e-04 | 1.670e-07 | 4.693e-02 |
| s_isl | 1.321e-03 | 3.009e-03 | 2.669e-07 | 5.369e-02 |
| p_opt_cont | 2.657e-01 | 2.520e-01 | 1.420e-01 | 4.610e-01 |
| p_opt_isl | 8.560e-02 | 6.800e-02 | 4.000e-03 | 3.600e-01 |

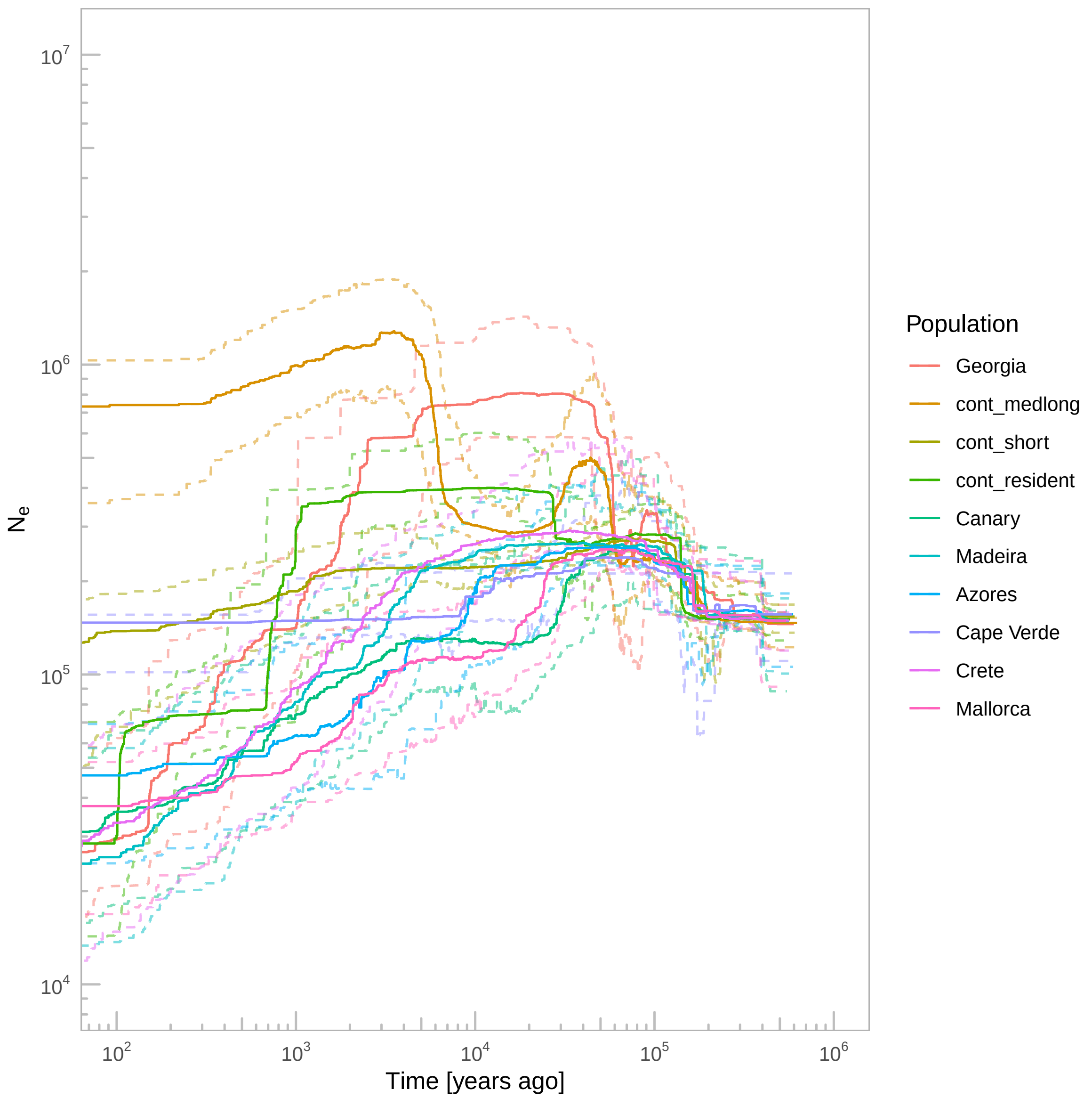


**Supplementary Figure 1:** Demography inference with `Stairway plot 2`.
